# Development and validation of a kidney renal clear cell carcinoma prognostic model relying on pyroptosis-related LncRNAs-A multidimensional comprehensive bioinformatics exploration

**DOI:** 10.1186/s40001-023-01277-2

**Published:** 2023-09-12

**Authors:** Chang Liu, Shuxin Dai, Hao Geng, Zhiwei Jiang, Xiangyu Teng, Kun Liu, Zhouting Tuo, Longfei Peng, Chao Yang, Liangkuan Bi

**Affiliations:** 1 Department of Urology, The Second Hospital of Anhui Medical University, Hefei, Anhui China; 2 Department of Urology, Peking University Shenzhen Hospital, Shenzhen, China

**Keywords:** Comprehensive bioinformatics, Long noncoding RNA, Kidney renal clear cell carcinoma, Pyroptosis, Immune response

## Abstract

**Background:**

Renal cell carcinoma (RCC) is a malignant tumour that may develop in the kidney. RCC is one of the most common kinds of tumours of this sort, and its most common pathological subtype is kidney renal clear cell carcinoma (KIRC). However, the aetiology and pathogenesis of RCC still need to be clarified. Exploring the internal mechanism of RCC contributes to diagnosing and treating this disease. Pyroptosis is a critical process related to cell death. Recent research has shown that pyroptosis is a critical factor in the initiation and progression of tumour formation. Thus far, researchers have progressively uncovered evidence of the regulatory influence that long noncoding RNAs (lncRNAs) have on pyroptosis.

**Methods:**

In this work, a comprehensive bioinformatics approach was used to produce a predictive model according to pyroptosis-interrelated lncRNAs for the purpose of predicting the overall survival and molecular immune specialties of patients diagnosed with KIRC. This model was verified from multiple perspectives.

**Results:**

First, we discovered pyroptosis-associated lncRNAs in KIRC patients using the TCGA database and a Sankey diagram. Then, we developed and validated a KIRC patient risk model based on pyroptosis-related lncRNAs. We demonstrated the grouping power of PLnRM through PCA and used PLnRM to assess the tumour immune microenvironment and response to immunotherapy. Immunological and molecular traits of diverse PLnRM subgroups were evaluated, as were clinical KIRC patient characteristics and predictive risk models. On this basis, a predictive nomogram was developed and analyzed, and novel PLnRM candidate compounds were identified. Finally, we investigated possible medications used by KIRC patients.

**Conclusions:**

The results demonstrate that the model generated has significant value for KIRC in clinical practice.

**Supplementary Information:**

The online version contains supplementary material available at 10.1186/s40001-023-01277-2.

## Background

There are approximately 210,000 new instances of renal cell carcinoma (RCC) diagnosed each year worldwide, accounting for between 2 and 3% of all tumour cases. The most prevalent kind of RCC is kidney renal clear cell carcinoma, which it accounts for approximately 85% of all RCC cases. Individuals diagnosed with KIRC almost always have poor prognosis, affecting both their health and life [1]. Patients with early-stage kidney cancer can be treated effectively with surgery. Nonetheless, after major surgery, approximately 30% of patients experience recurrence or metastasis, with poor prognosis for overall survival (OS) [2]. Typically, metastatic KIRC, a more advanced form of kidney cancer, is not entirely curable and has a short median survival. In recent years, immune checkpoint inhibitors, a new type of tumour therapy, have benefited some kidney cancer patients, especially PD-L1 and PD-1 inhibitors [3]. In reality, immunotherapy has a poor overall response rate of approximately 33%, and a significant minority of kidney cancer patients do not respond to this treatment [4, 5]. One explanation for the ineffectiveness of therapy, as shown by several study findings, is that some kidney cancer patients have limited sensitivity to immunosuppressive drugs; another cause is drug resistance and not the tumour per se. Consequently, to increase the chances of survival among patients with kidney cancer, it is crucial to investigate the molecular mechanisms responsible for the onset and development (as referred to as O and D) of this disease.

A growing number of studies have shed light in recent years on the impact of pyroptosis on the O and D of malignancies. According to conventional wisdom, pyroptosis describes the cell necrosis caused by certain bacterial invasions carried out by the proteinase caspase-1, which is specific for the amino acid cysteinyl aspartate. After the identification of inflammatory compounds in 2002 and discovery of nonclassical inflammasomes in 2011, pyroptosis has been regarded as a mechanism related to cell death and inflammatory bodies [6–8]. It has never been thought that caspase cleavage causes pyroptosis. Nevertheless, some research shows that the ability to cause pyroptosis may be achieved by expression of the N-terminal domain of Gasdermin D (GSDMD) or another Gasdermin [9–12]. In addition, activation of caspase is not necessary for pyroptosis. For instance, granzyme A and B act as upstream molecules of GSDMB and GSDME to cleave them, which is irrelevant to the action of caspase [13, 14]. Currently, pyroptosis has been redefined as apoptosis mediated by GSDM proteins. Pore-forming effector proteins comprise the GSDM family. For pyroptosis to occur, these proteins must first be cleaved and then have the capacity to create holes in the cell membrane [9, 15, 16].

An RNA that is 200 bp in length or more is referred to as long noncoding RNA (lncRNA). While lncRNAs are abundant in the cytoplasm and nucleus, they do not encode proteins [17]. The process of gene expression involves "noise", which has previously been thought to be lncRNAs [18]. The reality that synthesis of lncRNAs is comparable to that of coding genes was shown by DERRIEN et al. [19]. Splicing patterns, exon/intron organization, and histone modifications have all been discovered to be similar. Coding genes produce lncRNAs [20]. Presently, research suggests that lncRNAs play a critical role in the O and D stages of kidney cancer. OSRC-2 kidney cancer cells were found to overexpress the oncogene BCL-W protein, and WANG [21] discovered that the more aggressive the cancer cells were, the more lncRNA-RP11-436H11.5 was overexpressed. After we treated these cells with the inhibitor ATB-737, the tumour cells became less aggressive, and the inhibitory effect became more distinct when the concentration of ATB-737 was increased. The content of the lncRNA GIHCG was clearly greater in 46 RCC patient tissue and plasma samples than in normal tissues (P < 0.01), according to an analytical investigation by HE et al. [22].In addition, there is increasing evidence for the pre-target value of lncrnas in aging and aging-related diseases [23].

LncRNAs regulate proteins related to pyroptosis signalling through downstream pathways. MALA T1 may considerably raise NLRP3 levels by upregulating production of ELA VL1 proteins, which would further cause pyroptosis to develop in renal cells and damage in diabetic nephropathy model mice. Patients with uric acid kidney disease have increased expression of ANRIL, an opposing lncRNA at the INK4 locus. One study found that ANRIL might activate BRCC3, leading to production of NLRP3 and IL-1β/18, which is linked to renal disorders [24, 25]. Yi et al. shed further light on the processes involved in the elevation of NLRP3 expression triggered by lncRNA Gm4419 and subsequent release of IL-1β. Their research showed that in mouse mesangial cells cultivated with high glucose, NF-κB (p50) acts as a promoter of the NLRP3 inflammasome [26]. LncRNAs might be considered possible biomarkers of kidney diseases because they often have a favourable correlation with pyroptosis in kidney diseases.

The prognosis of KIRC was examined in this work using bioinformatics tools to assess the impact of pyroptosis-related lncRNAs. In addition, the application potential of a constructed prognostic model in tumour immunity and drug response was explored. Moreover, the accuracy of this model was verified in a separate cohort.

## Materials and methods

### Raw data

We used API v3.0.0 to download KIRC patient TCGA database mutation information, corresponding clinical data, and transcriptome RNA-seq data available at https://portal.gdc.cancer.gov (Release date: October 29, 2021). We used expression profile information from 91 RCC samples in the ICGC database (https://dcc.icgc.org/) as our validation cohort.

### pyroptosis-related prognostic LncRNA identification

According to Additional file 1: Table S1, 52 genes relevant to pyroptosis were gathered from earlier research and papers. Using a screening process based on gene annotation, 2876 lncRNAs were ultimately found in the TCGA cohort. The association study between 52 genes and lncRNAs associated with pyroptosis was conducted using the Pearson correlation coefficient. We identified lncRNAs associated with pyroptosis based on their absolute correlation coefficient (> 0.4) and *P*-value (< 0.001). As a consequence, 576 lncRNAs associated with pyroptosis were examined. After that, we performed univariate Cox regression analysis for OS, choosing lncRNAs with and without value in the process. P-values < 0.05 were used to determine whether lncRNAs are related to prognosis. Overall, 295 lncRNAs of prognostic relevance were found to be linked to pyroptosis.

### Construction and evaluation of the prognostic model

The training set served as the basis for construction of the prognostic model, and the test set and the whole TCGA set were used to evaluate the model's prediction performance. The training set was analyzed using multivariate Cox regression, revealing six pyroptosis-related lncRNAs. The model, named "PLnRM", uses the following formula to calculate the risk score: risk score = coef (lncRNA1) × expr (lncRNA1) + coef (lncRNA2) × expr (lncRNA2) + …… + coef (lncRNAn) × expr (lncRNAn). In this equation, "coef" represents coefficients. Specifically, "coef (lncRNAn)" refers to the survival-related coefficient of lncRNAs, and "expr (lncRNAn)" refers to expression of lncRNAs.

### Functional analysis

To verify differentially expressed genes (DEGs), we carried out Gene Ontology (GO) analysis using R-pack cluster profilers. Since *P* < 0.05 shows that functional annotations are enriched, this threshold is defined by the p-value.

### Analysis of the model for immunotherapeutic therapy

We evaluated and calculated abrupt change data using maftools of the R package, and we estimated the tumour mutation burden (TMB) by tumour-specific mutation genes. In addition, we used tumour immune dysfunction and exclusion (TIDE) computation to estimate immunotherapy response probability [27].

### PCA with Kaplan‒Meier survival analysis

Six pyrogenic lncRNAs, as well as genes and lncRNAs associated with pyroptosis, were studied by examining expression patterns [28]. In addition, differences in OS between the two models were assessed using Kaplan‒Meier survival analysis.

### Investigation of possible PLnRM-targeting compounds for therapeutic use

Using the GDSC website, we were able to identify the semimaximum inhibitory concentration (IC50) of medications to locate therapeutic chemicals for KIRC patients and enhance clinical effectiveness. To calculate the IC50 for KIRC patients, we employed a pRophetic formula found in the R package.

### PLnRM independence

Cox regression models, including single- and multiple-variable models, were utilized to show that individuals with KIRC do not have a prognostic pattern that is distinct from or unrelated to other clinical characteristics [29].

### Establishing and verifying the predictive nomogram

We created a graph throughout the procedure to predict OS at 1, 3, and 5 years for KIRC patients to improve practicability and make the findings clear. We use the Hosmer‒Lemeshow test to demonstrate the consistency of the corrective curve.

### Molecular and immune characteristics and ICI therapy in PLnRM risk groups

During signalling pathway analysis, we first analyzed differential expression of gene samples with R's limma package to assess samples with high (n = 285) and low (n = 245) PLnRM scores. Enrichment analysis was conducted to identify signalling pathways in which differentially expressed genes are involved using the gene set enrichment analysis (GSEA) approach. The KEGG and HALLMARK gene sets were used, along with the cluster profile package of R. A significance threshold of P < 0.05 and FDR < 0.25 was applied. For a few common gene sets, we next conducted single-sample GSEA (ssGSEA). Differences in patient survival were then explored using Kaplan‒Meier survival curves. The cBioPortal database provides information on gene changes for gene mutation research. Using the R package Maftools, we examined the quantity and quality of mutations in two PLnRM risk groups. Correlation analysis was performed on expression of PD1, PDL1, CTLA4, BTLA, and CD24 between the two PLnRM risk groups. We imported expression data from 530 KIRC samples into CIBERSORT (https://cibersort.stanford.edu/). After 1000 iterations, we estimated the relative percentage of 22 categories of immune cells to identify their immunological features. Additionally, the matching proportions of 22 different kinds of immune cells as well as clinicopathological variables were compared between the two PLnRM risk groups, and the findings are shown in a landscape map. ssGSEA was conducted on specific gene signatures to better elucidate the immunological and molecular functions between the two PLnRM risk groups, and the resulting scores were also compared [30–33].

## Results

### Discovery of pyroptosis-associated LncRNAs in KIRC patients

**Fig. 1:**
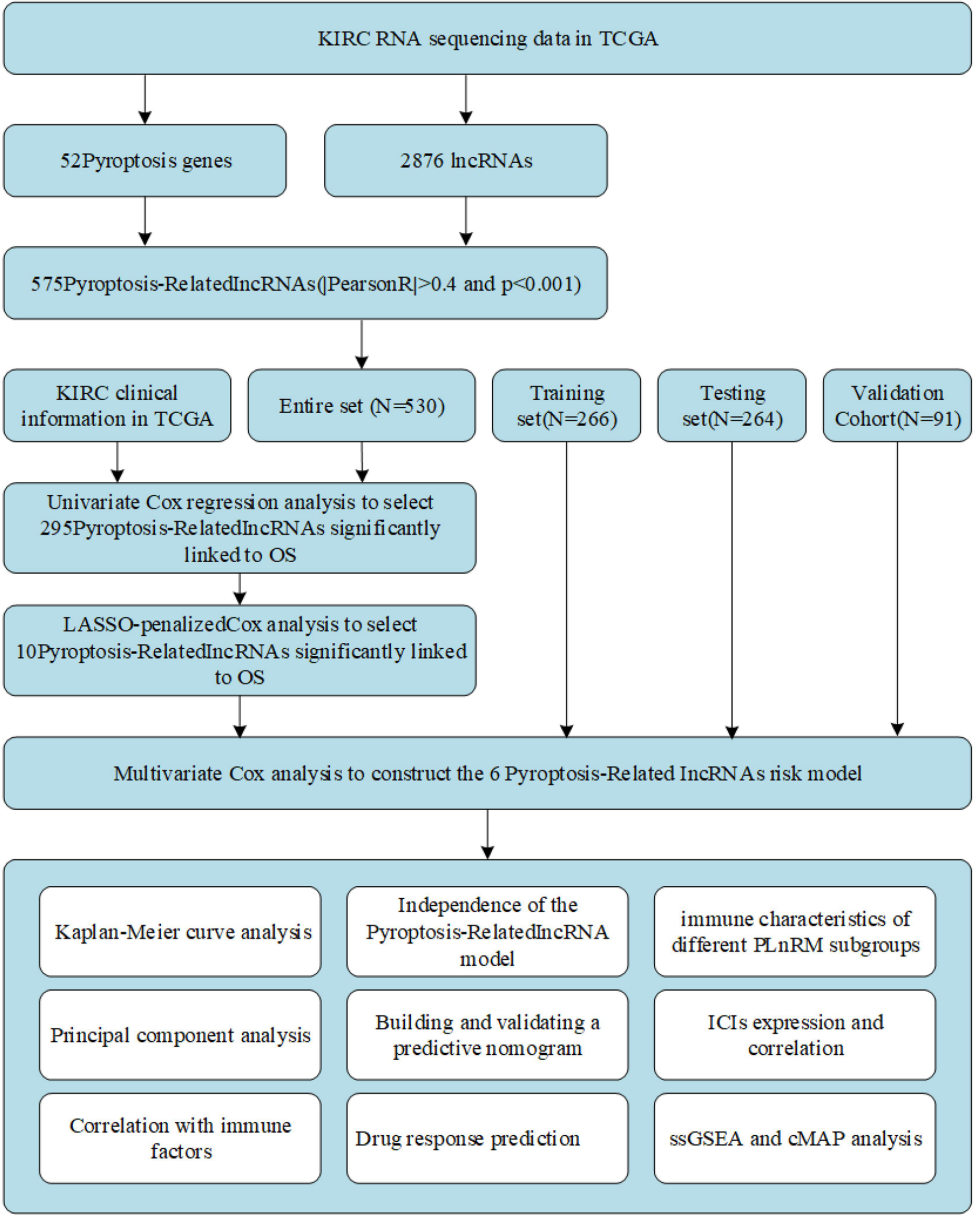
Comprehensive research workflow

**Fig. 2:**
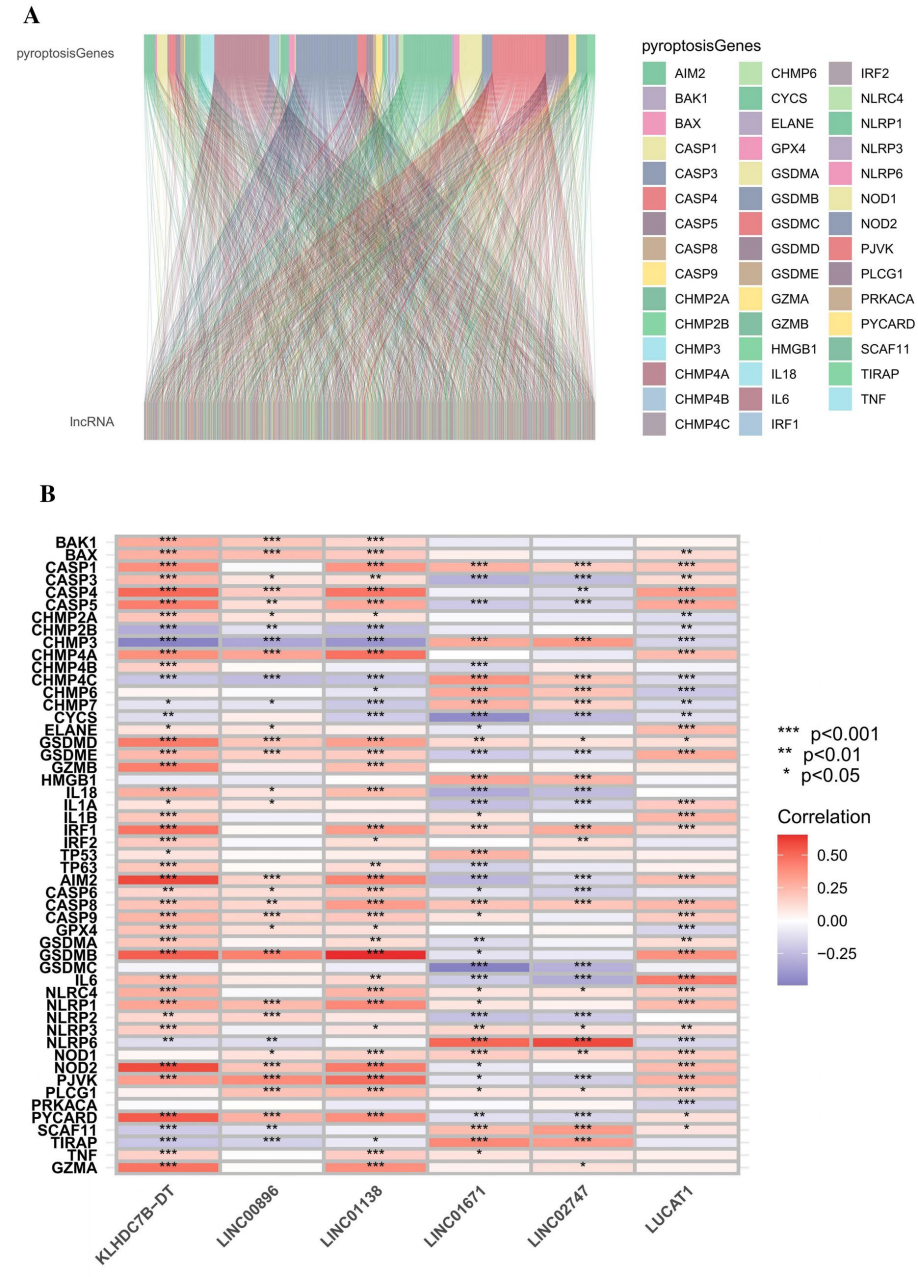
Discovery of lncRNAs associated with pyroptosis in KIRC patients. **A** Fifty-two pyroptosis genes and lncRNAs are represented by a Sankey diagram. **B** Heatmap depicting the relationship between 52 pyroptosis genes and 6 prognostic pyroptosis-associated lncRNAs

Figure 1 illustrates the precise procedure for building the risk model and subsequent analyses. The TCGA database was used to derive matrix expression of 52 pyroptosis genes and 2,876 lncRNAs. We classified pyroptosis-related lncRNAs as those strongly linked to at least one of the 52 pyroptosis genes (|Pearson *R*|> 0.4 and *P* < 0.001). The pyroptosis-lncRNA coexpression network is depicted using a Sankey diagram (Fig. 2A). The results revealed 576 lncRNAs to be associated with pyroptosis. Figure 2B shows the relationship between pyroptosis genes and pyroptosis-related lncRNAs in the whole TCGA dataset.

### KIRC patient risk model development and validation based on pyroptosis-related LncRNAs

**Fig. 3:**
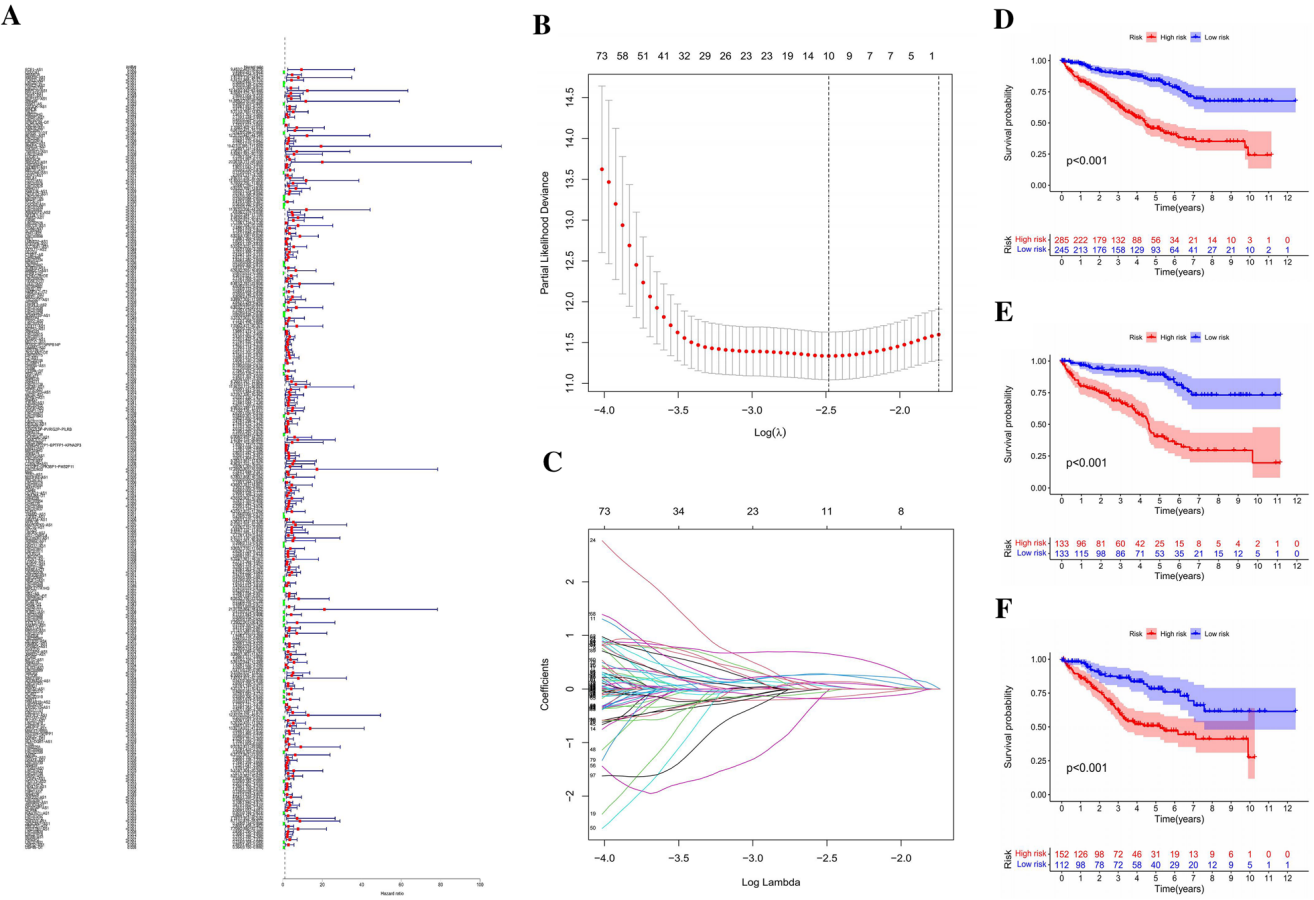
The risk model for KIRC patients based on pyroptosis-related lncRNAs. **A** The identified lncRNAs have a substantial correlation with clinical prognosis, as indicated by univariate Cox regression analysis. **B** The LASSO coefficient profile of ten OS-related lncRNAs and imaginary perpendicular lines were drawn at the value determined by tenfold cross-validation. **C** Tuning parameters (logλ) of OS-related proteins were selected for error curve cross-validation. At the ideal value, perpendicular fictitious lines were drawn in accordance with the minimum criteria and 1-se criterion. **D**–**F** OS Kaplan‒Meier survival curves for high- and low-risk patient populations (the entire TCGA, training, and test sets)

We conducted univariate Cox regression analysis on 2876 pyroptosis-related lncRNAs in the KIRC dataset included in the TCGA database to identify prognostic lncRNAs. The TCGA database contains 295 pyroptosis-related lncRNAs with a strong correlation with the OS of KIRC patients (Fig. 3A). Multiple regression analysis often uses LASSO-penalized Cox analysis. It may concurrently perform selection and regularization of variables in addition to improving the statistical model's capacity for and accuracy at making predictions. This technique has been used widely to choose the best features in data in high dimensions with little connection and a strong projected value to prevent overfitting. As a result, this technique can efficiently identify the best predictive markers and provide a prognostic indicator to predict clinical outcomes. The value of log l that ranks first is shown on the dashed perpendicular line with the least bias in segment probability. Hence, Fig. 3B and C shows that 10 lncRNAs associated with pyroptosis were chosen for further multivariate analysis. Based on this, autocephalous prognostic proteins were identified using multivariate Cox ratio hazard regression analysis. To create a risk model for assessing the prognosis of KIRC patients, we utilized 6 pyroptosis-related lncRNAs (listed in Additional file 2: Table S2) that were independently associated with OS in the training set (as shown in Additional file 3: Table S3).

**Fig. 4:**
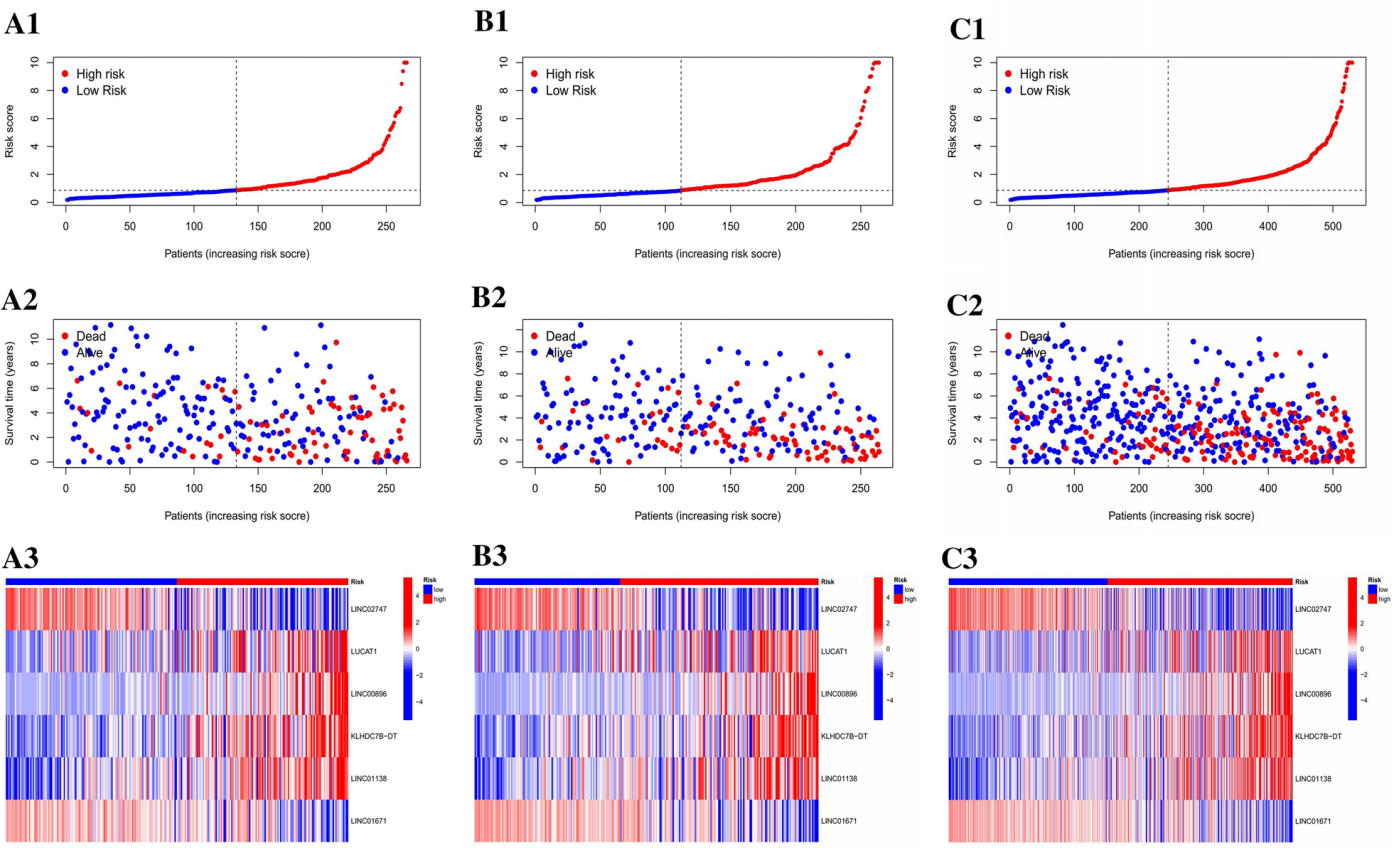
Assessment of the predictive value of PLnRM risk patterns in the TCGA dataset. **A**1 Distribution of PLnRM-based risk scores. **A**2 Both groups exhibit distinct survival status and duration patterns. **A**3 The heatmap resulting from clustering analysis displays expression levels of PLnRM in each patient. **B**–**C** Relevant findings from both the training and test sets

We conducted Kaplan‒Meier survival analysis on KIRC samples, dividing them into low-risk and high-risk groups using median prognostic risk scores. Figure 3D–F displays the survival status of patients in both groups across the full TCGA set, training set, and test set. The results showed a significant (P < 0.001) difference in outcomes between the high-risk and low-risk groups. Figure 4 A1 and 4A2 displays the patient survival status, survival time, and risk level distribution for both groups. The six lncRNAs associated with pyroptosis and their relative expression standards in each patient are shown in Fig. 4A3.

We used the same formula to calculate each patient's risk score in both the training and test sets. This allowed us to assess how effective our model is at predicting prognosis. Figures 4B, C depict expression of pyroptosis-related lncRNAs in the training set (Fig. 4B1–B3) and test set (Fig. 4 C1–C3). Additionally, they illustrate the distribution of risk grades, survival status patterns, and survival times (Fig. 4C1–C3).

**Fig. 5:**
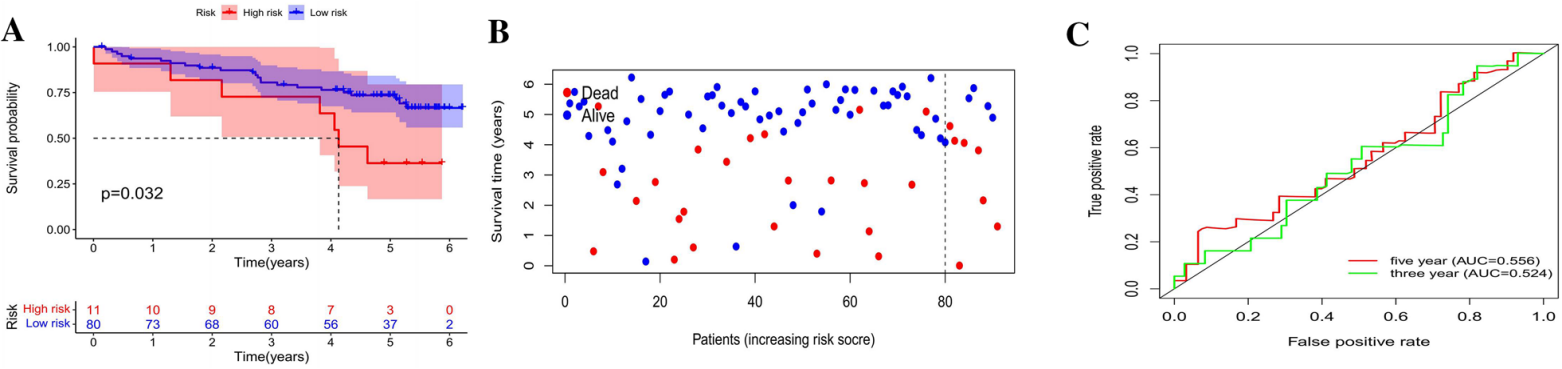
Independent cohort validation. **A** OS Kaplan‒Meier curves for high-risk and low-risk patient groups. **B** Model-based risk score distribution for m6A-related lncRNAs in the validation set. **C** ROC curves for clinical characteristics (3 and 5 years)

To confirm the precision and applicability of the model, we verified expression profile data for 91 RCCs in the ICGC database. The results demonstrate the model's continued effectiveness in predicting survival time, particularly long-term survival (Fig. 5).

We compared differences in OS between low- and high-risk groups across the entire TCGA dataset, stratifying by common clinicopathologic characteristics. The low-risk group's OS remained superior to that of the high-risk group across subgroups categorized by age, sex, stage, and grade (see Additional file 4: Figure S1).

### Validation of the PLnRM grouping capability through PCA

**Fig. 6:**
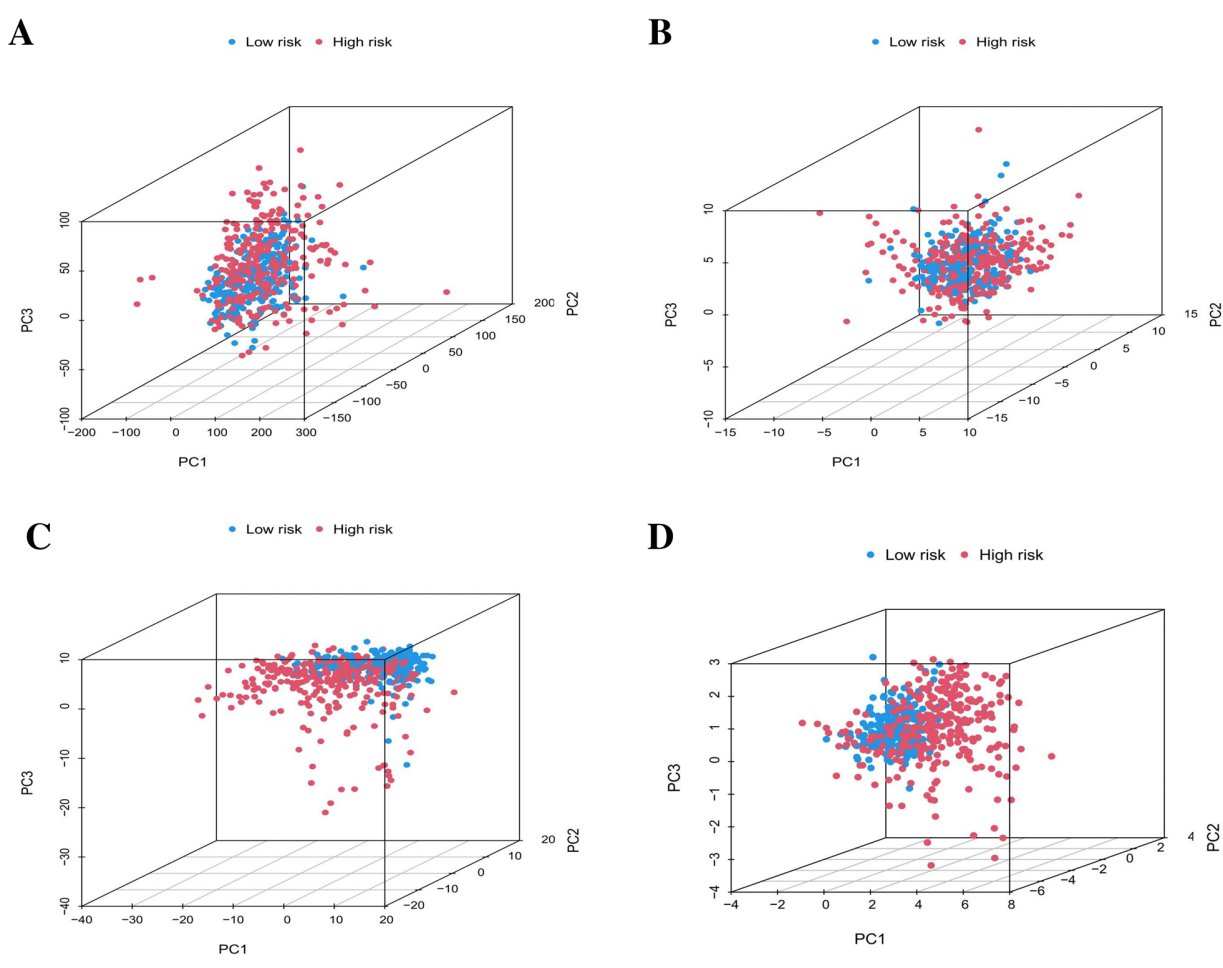
We conducted principal component analysis on four different datasets: **A** entire gene expression profiles, **B** 52 pyroptosis genes, **C** 576 lncRNAs associated with pyroptosis, and **D** a risk model using six pyroptosis-related long noncoding RNAs from the entire TCGA dataset

Comprehensive gene expression profiles, 52 pyroptosis genes, lncRNAs related to pyroptosis, and a risk model separated by lncRNA expression profiles all contributed to this model, which was then subjected to principal component analysis (PCA) to validate the distinction between low-risk and high-risk groups (Fig. 6A–D). As shown in Fig. 6A–C, there was absolutely no uniformity in the distribution of the high-risk and low-risk groups. Nonetheless, the findings from this model showed that the two groups’ distributions were significantly different (Fig. 6D). Based on these findings, it seems that there is a difference in the prognostic signature between the two groups.

### PLnRM to assess the tumour immune microenvironment and response to immunotherapy

**Fig. 7:**
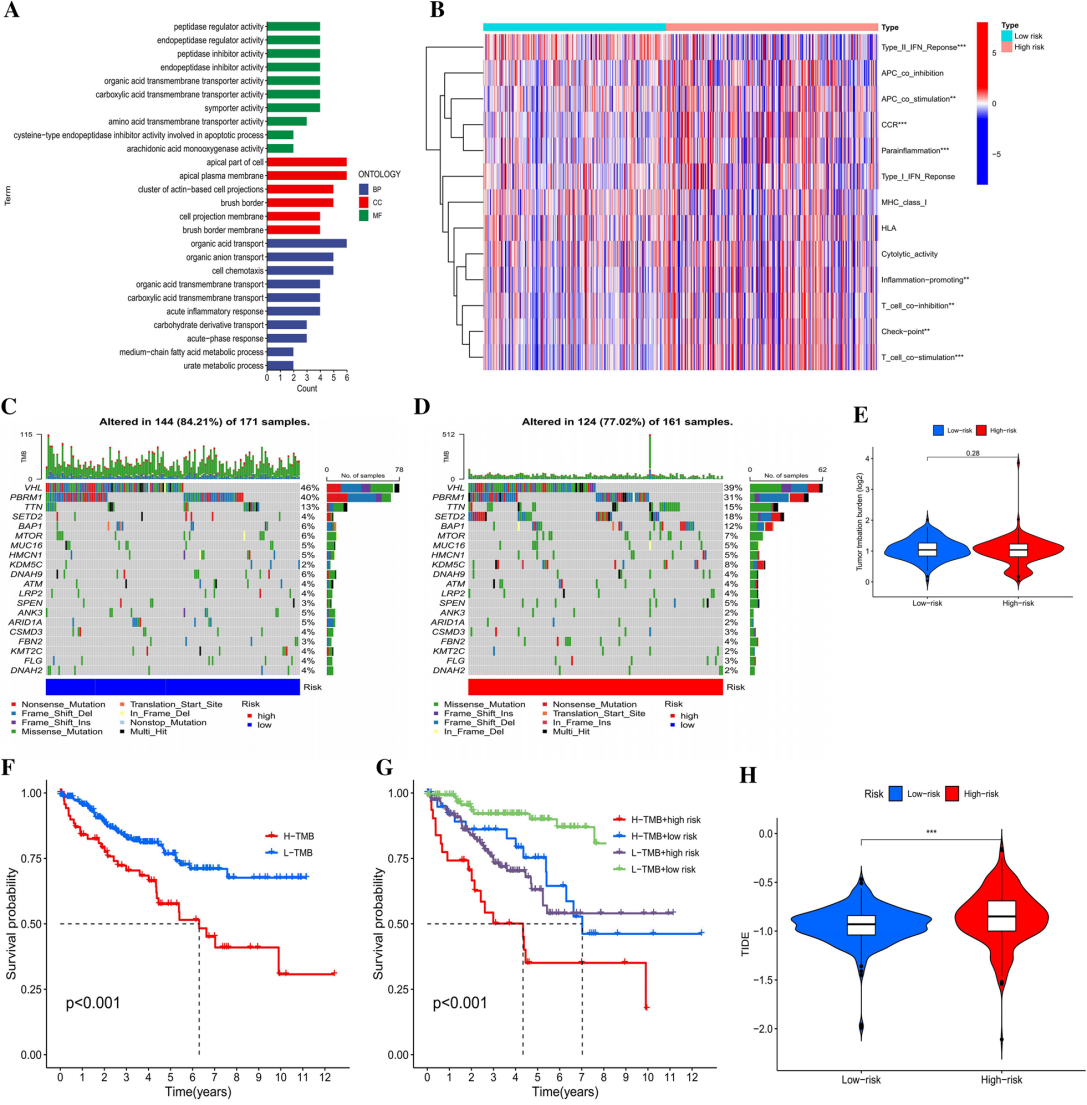
Estimation of the tumour immune microenvironment and immunotherapy response using PLnRM for the full TCGA dataset. **A** GO enrichment analysis. **B** The specified requirements for each patient's immunity index. **C**, **D** The waterfall plot shows gene mutation data for high-risk and low-risk groups, specifically highlighting genes with high mutation rates. **E** TMB was different between the patients in the two groups. **F**–**G** Patient mutation status (high or low) and their PLnRM were taken into account for the Kaplan‒Meier analysis of their OS. **H** Differences in TIDE prediction for patients between the two groups

PLnRM was utilized to analyze the enrichment and activity levels of various immune cells, pathways, and activities in 530 KIRC patients. The findings showed that most immunological indicator expression levels between the two groups were significantly different (Fig. 7B). A GO enrichment study was performed to explore the putative molecular mechanisms related to PLnRM. Regarding BP, the model genes are related to a variety of biological functions relevant to the immune system (Fig. 7A). The relationship between PLnRM and the effectiveness of immunotherapy was, therefore, investigated. PLnRM can predict TIDE by indicating that the high-risk group will be more responsive to immunotherapies than the low-risk group, as hypothesized (Fig. 7H). In addition, maftools of the R package were used to investigate and assemble the data associated with mutations, and the consequences of mutation were used for stratification. Figure 7C and D displays the top 20 driver genes that show the greatest frequency change across all categories. TMB scores were then computed on the basis of the TGCA somatic mutation data. The findings indicate no significant difference in tumour mutation between the high-risk and low-risk groups, as shown in Fig. 7E. Kaplan‒Meier survival analysis of TMB was conducted using tumour tissues. The results (Fig. 7F) indicated that the low-mutation group had a higher chance of surviving than the high-mutation group. Furthermore, it was found that the prognosis for those in the high-mutation and high-risk groups was worse than that for those in the low-mutation and low-risk groups. Even when there were two groups with either a high or low mutation risk (Fig. 7G), individuals in the high-risk group still had a poorer prognosis than those in the low-risk group. This risk model seems to be reliable and stable based on these findings and our earlier findings.

### Immunological and molecular traits of diverse PLnRM subgroups

**Fig. 8:**
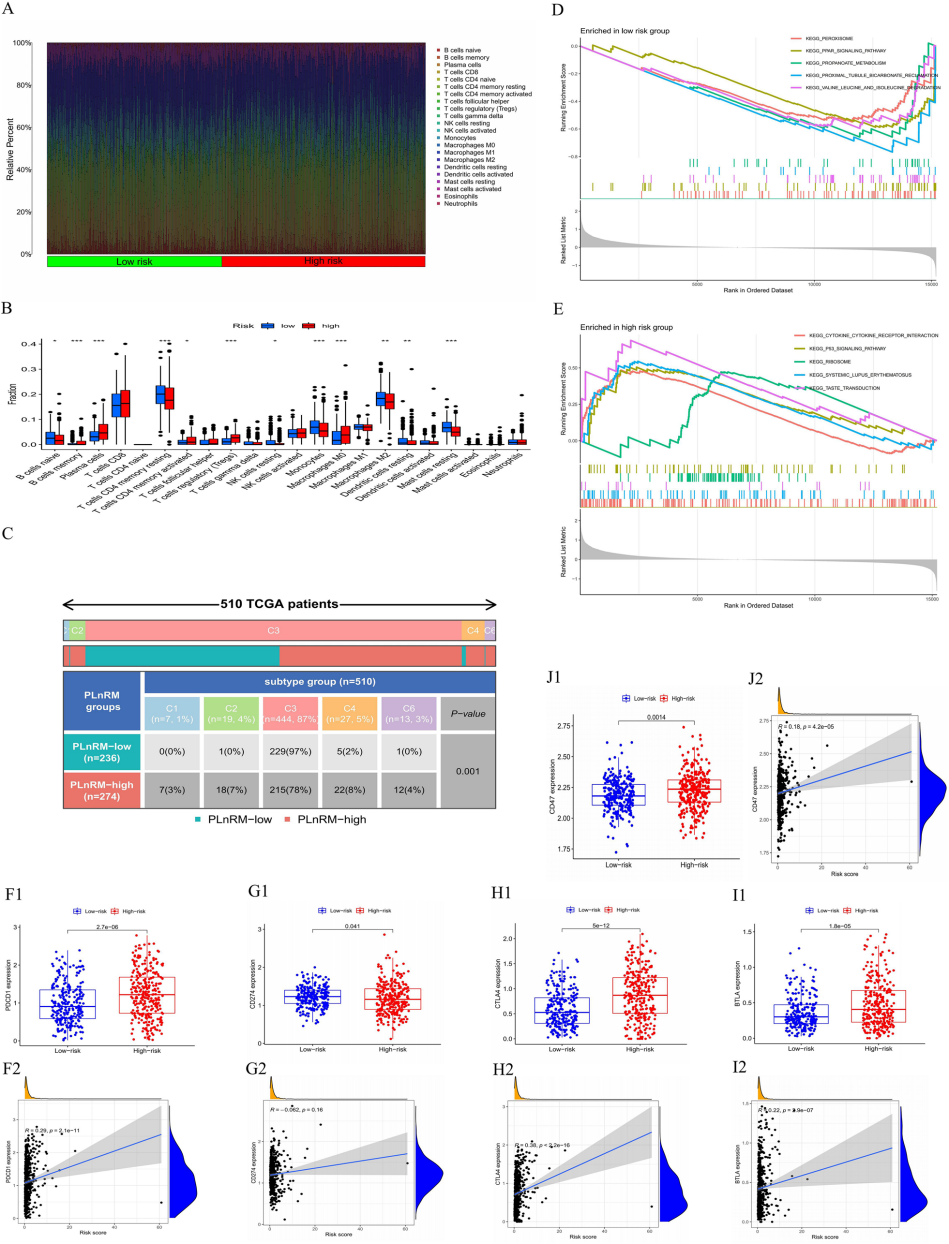
Molecular and immune features of several PLnRM subgroups. **A** A bar graph depicting the relative proportion of 21 immune cells infiltrating tumours in the high-risk and low-risk groups. **B** The violin plot illustrates the disparity in the proportions of each type of immune cell in the two risk groups. **C** Qualities associated with the immune system. **D** and **E** Gene set enrichment analysis using the Kyoto Encyclopedia of Genes and Genomes (KEGG) for the low-risk group and high-risk group. **F**–**J** The two groups differ in expression and associations of common immunological checkpoints

The Wilcoxon test was used to examine the immune cell distribution in various PLnRM subgroups with the intention of examining the immune cell composition in various PLnRM subgroups. According to the results, the group with PLnRM-high risk had a higher prevalence of regulatory T cells (Tregs), activated memory CD4 T cells, CD8 T cells, memory B cells (MBCs), and macrophages (M0). Resting dendritic cells, macrophages (M2), resting NK cells, gamma delta (γδ) T cells, T follicular helper (Tfh) cells, resting memory CD4 T cells, plasma cells, naïve B cells, and activated dendritic cells were more prevalent in the PLnRM-low risk group, as shown in Fig. 8A, B. Figure 8C displays traits connected to the immunological landscape.

The gene sets enriched in various PLnRM subgroups were determined using GSEA. The P53 signalling pathway, ribosome, cytokine receptor interaction, systemic lupus erythematosus, and taste transduction pathways were enriched in the gene sets for samples with significant levels of PLnRM (Fig. 8D). However, according to Fig. 8E, the PLnRM-low samples showed gene sets that were enriched in pathways related to tumour metastasis (P < 0.05, FDR < 0.25). To further investigate how the two groups' responses to immunotherapy differed, variations in immune checkpoint expression and correlation were evaluated. As shown in Fig. 8F–J, CD47, B- and T-lymphocyte attenuator (BTLA), cytotoxic T lymphocyte-associated antigen (CTLA), PD ligand 1 (PD-L1), and programmed cell death (PD-1) were all higher in the high-risk group.

### Clinical KIRC patient features and evaluation of the predictive risk model

**Fig. 9:**
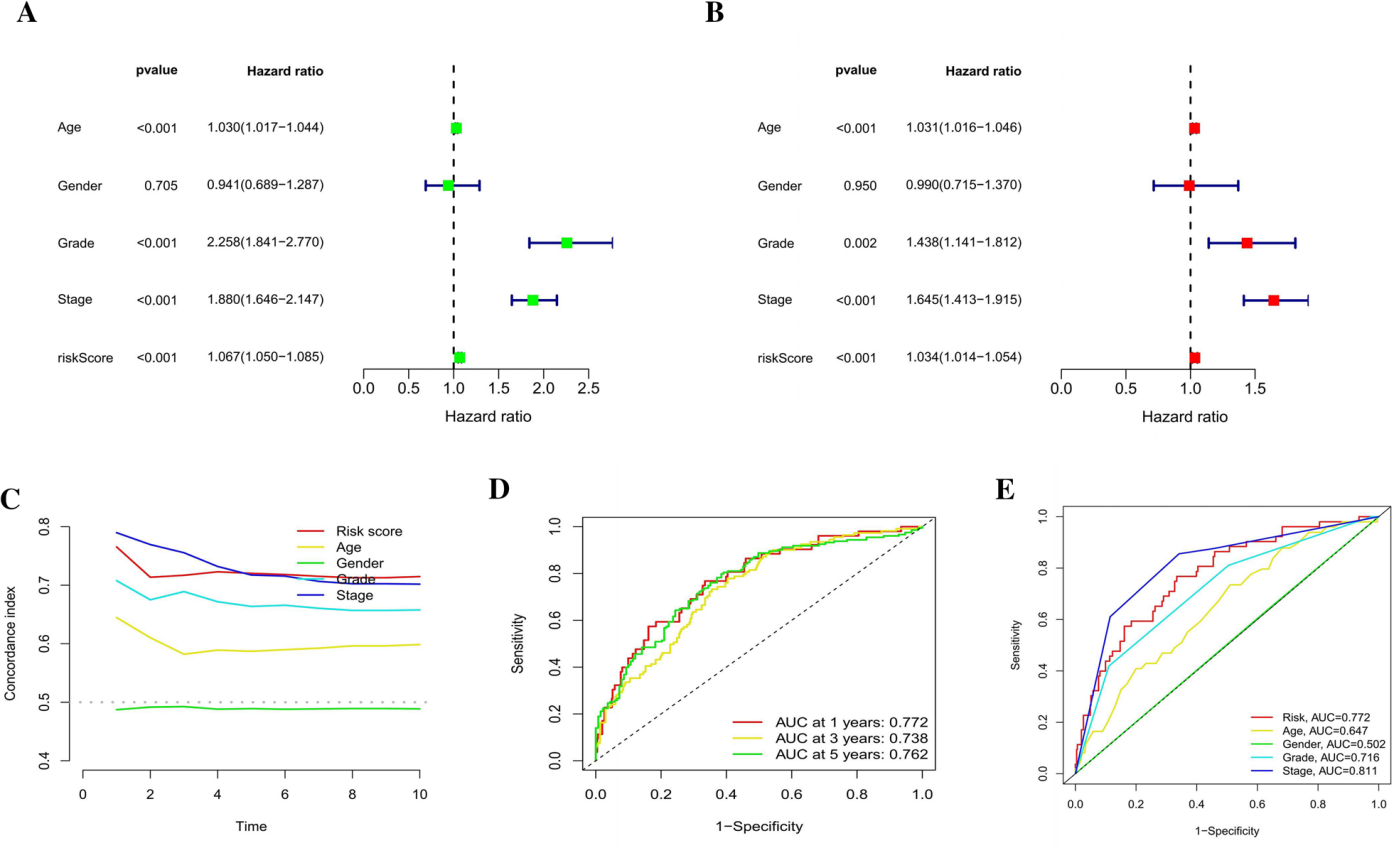
Evaluation of the clinical characteristics of KIRC as well as the prognostic risk model using the whole TCGA dataset. **A**–**B** Assessments of the clinical features and risk scores linked with OS using both univariate and multivariate methods. **C** Concordance indicators for the risk score and clinical features. **D**–**E** ROC curves of various clinical parameters and risk scores

This investigation utilized univariate and multivariate Cox regression analyses to determine whether the risk model incorporates independent prognostic factors for KIRC. Multivariate Cox regression analysis showed a hazard ratio (HR) of 1.067 and a 95% confidence interval (CI) of 1.050–1.085, with a significance level of P < 0.001 (Fig. 9A). In univariate Cox regression analysis, the odds ratios (HRs) were found to be 1.034, with a corresponding CI of 1.014–1.054, and significant results at P < 0.001 (Fig. 9B). The findings support the notion that the risk model can predict prognosis regardless of other clinical variables. We evaluated the distinctiveness and sensitivity of risk scores in predicting KIRC patient outcomes using the concordance index and area under the receiver operating characteristic (ROC) curve (AUC), as shown in Fig. 9D, E. Longer follow-up periods resulted in a greater concordance index of risk ratings, which ultimately trumped other clinical criteria and increased risk severity. Thus, the prognosis of KIRC patients may be accurately predicted using this model's risk score (Fig. 9C). Moreover, the AUC representing the risk level rose to a point where it exceeded that of the vast majority of other clinicopathological characteristics. Our results tended to support the idea that PLnRM is a viable option for incorporation into the predictive risk model that is currently being used for KIRC patients (Fig. 9C).

### Development and analysis of the predictive nomogram

**Fig. 10:**
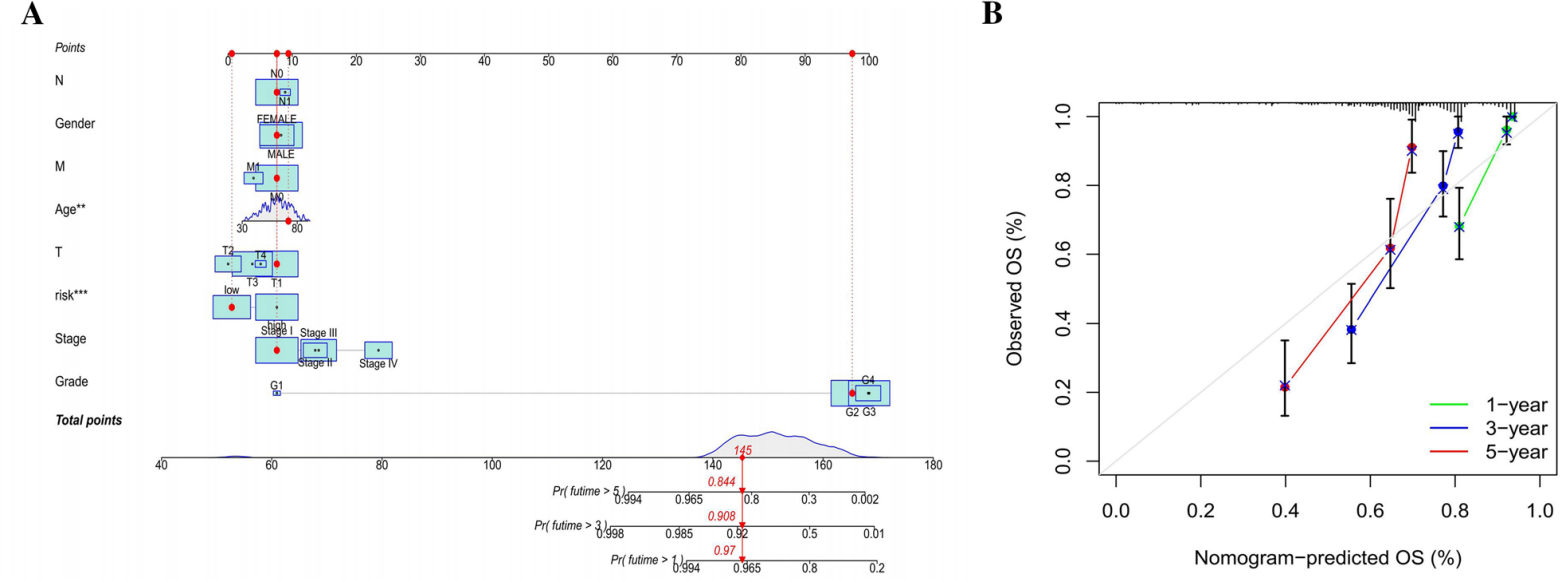
A predictive nomogram was constructed and evaluated. **A** The nomogram predicts the probability of 1-, 2-, and 3-year overall survival. **B** The nomogram calibration plot was created using the OS probabilities for 1, 2, and 3 years

The 1-, 2-, and 3-year OS of KIRC patients can be predicted using a nomogram that takes into account both risk levels and clinical risk factors. The nomogram revealed that the prediction model's risk level has high predictive power when compared with clinical parameters (Fig. 10A). The 1-, 2-, and 3-year OS observation and prediction rates were all shown to be in good agreement by means of appropriate diagrams (Fig. 10B).

### Identification of novel PLnRM candidate compounds

We used the prediction approach to evaluate treatment response for KIRC patients by analyzing the IC50 of each sample in Genomics of Drug Sensitivity in Cancer (GDSC). Our goal was to identify potential medications for PLnRM. There were 110 discovered compounds in all, and the predicted IC50 values between the two groups varied significantly. Partially sensitive chemicals are shown in Additional file 5: Figure S2.

### Investigation of possible medicines for patients with KIRC

Several possible therapeutic medications for KIRC were evaluated using the connection map (CMAP) database to elucidate a new therapy plan for the disease. The top 10 closely related molecules of medications were chosen to depict the three-dimensional structure (Additional file 6: Figure S3).

## Discussion

The third most common tumour in the urinary system by incidence, RCC, is a kind of cancer that develops from the epithelium that lines the renal tubules, and its prevalence is growing [34]. Although surgical resection is the most effective treatment for RCC, many patients receive their diagnosis in the middle or late stage. Furthermore, these tumours are unresponsive to chemotherapies, immunotherapies, and radiotherapies. Targeted treatments may also lead to temporary drug resistance. RCC patients often have poor prognosis as a result [35, 36]. RCC is influenced by a number of different variables that may impact its onset and progression, as well as by a number of different genes. Another significant contributor to RCC is abnormal alterations in the network that controls gene expression [37]. Control of gene expression is influenced by various factors, including the level of genes and their regulation at different stages, such as transcription, translation, and protein degradation. Understanding lncRNA roles and identification allows for new perspectives on how gene expression is regulated.

Pyroptosis is a type of cell death that involves rapid rupture of cell membranes, resulting in release of cellular contents and proinflammatory substances such as interleukin (IL), IL-1β, and IL-18. This process is characterized by cell swelling and the formation of large bubbles in the cytoplasm [38, 39]. The consequences of pyroptosis on several inflammation-related disorders, such as heart disease, sepsis, diabetes, nephropathy, and atherosclerosis, are mostly due to release of these distinctive inflammatory factors [40–42].

In recent years, there have been many breakthroughs in understanding the mechanisms, molecules, and pathways related to pyroptosis. Pyroptosis has been shown to be related to the incidence, growth, prognosis, and treatment of a number of cancers [43, 44]. In addition, pyroptosis has been verified to participate in chimeric antigen receptor T-cell immunotherapy (CAR-T therapy), cytokine release syndrome (CRS), and chemotherapy [45–47]. In macrophages, certain factors induce the release of DNA to trigger the cGAS-STING induced IFN response, thereby regulating pyroptosis and inflammation, which provides further evidence for the regulation of pyroptosis [48–50]. Exploring the mechanism of pyroptosis and its correlation with tumours from a comprehensive perspective is conducive to broadening our understanding of tumours, which might indicate a new direction for cancer therapy.

The process of pyroptosis may be directly regulated by lncRNAs, as previously indicated, in addition to having an indirect effect. LncRNAs have been shown to directly control pyroptosis, according to recent studies. For example, it was found that the lncRNA Neat1 stabilizes mature caspase-1 tetramers (p20: p10)2 and (p33: p10)2 in mouse bone marrow-derived macrophages (BMEMs) treated with flagellin and poly(I:C) after induction with LPS. This study found that lncRNA directly binds to pro-caspase-1, promoting assembly of NLRP3 and AIM2 inflammasomes. Additionally, it was discovered that lncRNAs play a regulatory role in the pyroptosis signalling pathway affecting the inflammasome. Hence, a KIRC predictive model produced using lncRNAs associated with pyroptosis would be valuable.

In this work, we built an independent prognostic model based on lncRNAs associated with pyroptosis in KIRC, considering the functions of both, and whether there are any medications that may be useful for treating KIRC is thus being explored. It is possible to investigate the prognostic role of pyroptosis-related lncRNAs using the 576 pyroptosis-related lncRNAs in the TCGA database. The TCGA database findings show that 10 pyroptosis-related lncRNAs have predictive significance and that a model including these lncRNAs may be useful to predict the overall survival rate in KIRC patients after utilizing these 6 lncRNAs. The median prognostic risk score was used to classify KIRC patients as having high or low risk. The high-risk group had poorer prognosis, as indicated by outcomes. Multivariate Cox regression analysis revealed that the lncRNA model associated with pyroptosis is an autologous risk factor for OS. According to the findings of ROC analysis, this model is superior to the majority of common clinical characteristics in predicting the overall survival of individuals with KIRC. In addition, a nomogram was shown to illustrate the degree to which the observed and predicted OS rates correspond over the first, third, and fifth years of follow-up. The prediction rates for the first, third, and fifth years all agreed quite well. The risk model built using 10 pyroptosis-associated lncRNAs that are independently related to KIRC OS has greater accuracy. This prediction model can help to identify new biomarkers for future research.

The TIDE method is also used to predict the probability of an immunotherapeutic response, revealing that the high-risk group had a higher immune response rate than the low-risk group. This suggests that immune-related medications may be more effective in predicting outcomes for the high-risk group. This discovery may be applied to guidelines for immune-related medications.

Furthermore, the model was used to analyze the immunological and biochemical traits of various subgroups. According to the findings, there are some variations between the high-risk and low-risk groups in terms of immune cell enrichment and infiltration. While analyzing expression and connection of popular immune-related genes such as PD1 and PD-L1, significant differences in their expression between the high-risk and low-risk groups were found. Moreover, there was an inverse relationship between the risk score and expression of these genes.

This model was validated using datasets found in the TCGA database. The ICGC RCC dataset is integrated as an external cohort so that the accuracy of this model and its application may be evaluated. The findings of the survival research indicate that there is a significant gap between the high-risk and low-risk groups. As a result, our model is capable of accurately predicting the chances of survival for KIRC patients. With the help of AI and deep learning models, we can well utilize radio-genomics combined with the prediction model of KIRC reported in this paper [51].

The prognosis of KIRC patients is largely determined by the pathological stage and grade, yet tumours with the same clinical stage and grade may not always have the same prognosis [52]. Exploring more detailed and focused prediction signs or biomarkers is thus very important. The pyroptosis-related lncRNA model that was developed is intended to provide a novel strategy for predicting the prognosis of KIRC patients. These results offer a new approach to studying lncRNAs associated with pyroptosis and modification processes. Several approaches are used in this work to validate the new model, making it possible to choose and implement the best model. Due to the lack of external data verification, it may be presumed that this prediction model is appropriate.

However, this research still has several shortcomings. For example, some questions about the biological mechanism of lncRNAs associated with pyroptosis remain. Investigating lncRNA function and how lncRNAs interact with genes involved in pyroptosis is thus crucial. As a whole, the conclusions provide fresh perspectives for predicting KIRC patient survival and prognosis, which may help to shed light on the mechanism behind pyroptosis-related lncRNAs. As a result of the development of this immunotherapy-sensitive model, several preliminary medication candidates were also found that may be useful, impacting the therapeutic approach used for KIRC patients.

### Supplementary Information

**Additional file 1: Table S1.** Gene names of the 52 pyroptosis-related genes.

**Additional file 2: Table S2.** Names of the six lncRNAs in the model that are connected to pyroptosis.

**Additional file 3: Table S3.** In the risk model, the clinical characteristics are shared by the complete set, the training set, and the test set.

**Additional file 4: Figure S1.** Kaplan‒Meier curves show differences in overall survival between the two groups in the entire TCGA set, stratified by age, sex, tumour grade, and stage.

**Additional file 5: Figure S2.** Compounds with partial sensitivity.

**Additional file 6: Figure S3.** Three-dimensional structure of the top ten related drugs.

## Data Availability

The datasets presented in this study can be found in online repositories. The names of the repositories/repositories and accession number(s) can be found in the article/Supplementary Material.

## Abbreviations

RCC: Renal cell carcinoma
KIRC: Kidney renal clear cell carcinoma
lncRNAs: Long noncoding RNAs
OS: Overall survival
GSDMD: Gasdermin D
GO: Gene Ontology
TMB: Tumour mutation burden
TIDE: Tumour immune dysfunction and exclusion
GSEA: Gene set enrichment analysis
MBCs: Memory B cells
Tfh: T follicular helper
PCA: Principal component analysis
BTLA: B- and T-lymphocyte attenuator
HR: Hazard ratio
GDSC: Genomics of Drug Sensitivity in Cancer
IL: Interleukin
CRS: Cytokine release syndrome
BMEMs: Bone marrow-derived macrophages

## References

[1] Ljungberg B, Albiges L, Abu-Ghanem Y, Bensalah K, Dabestani S, Fernández-Pello S (2019). European association of urology guidelines on renal cell carcinoma: the 2019 update. Eur Urol.

[2] Finelli A, Ismaila N, Bro B, Durack J, Eggener S, Evans A (2017). Management of small renal masses: american society of clinical oncology clinical practice guideline. J Clin Oncol..

[3] Ge W, Song S, Qi X, Chen F, Che X, Sun Y (2023). Review and prospect of immune checkpoint blockade therapy represented by PD-1/PD-L1 in the treatment of clear cell renal cell carcinoma. Oncol Res.

[4] Motzer RJ, Escudier B, McDermott DF, George S, Hammers HJ, Srinivas S (2015). Nivolumab versus Everolimus in advanced renal-cell carcinoma. N Engl J Med.

[5] Zeng SY, Liu YF, Liu JH, Zeng ZL, Xie H, Liu JH (2023). Potential effects of *Akkermansia*
*Muciniphila* in aging and aging-related diseases: current evidence and perspectives. Aging Dis.

[6] Martinon F, Burns K, Tschopp J (2002). The inflammasome: a molecular platform triggering activation of inflammatory caspases and processing of proIL-beta. Mol Cell.

[7] Hersh D, Monack DM, Smith MR, Ghori N, Falkow S, Zychlinsky A (1999). The Salmonella invasin SipB induces macrophage apoptosis by binding to caspase-1. Proc Natl Acad Sci U S A.

[8] Brennan MA, Cookson BT (2000). Salmonella induces macrophage death by caspase-1-dependent necrosis. Mol Microbiol.

[9] Cookson BT, Brennan MA (2001). Pro-inflammatory programmed cell death. Trends Microbiol.

[10] Tsuchiya K (2021). Switching from apoptosis to pyroptosis: gasdermin-elicited inflammation and antitumor immunity. Int J Mol Sci..

[11] Zhang Z, Lieberman J (2020). Lighting a fire on the reef. Sci Immunol..

[12] Xu YJ, Zheng L, Hu YW, Wang Q (2018). Pyroptosis and its relationship to atherosclerosis. Clin Chim Acta.

[13] Pirzada RH, Javaid N, Choi S (2020). The roles of the NLRP3 inflammasome in neurodegenerative and metabolic diseases and in relevant advanced therapeutic interventions. Genes (Basel)..

[14] Zhang Z, Zhang Y, Xia S, Kong Q, Li S, Liu X (2020). Gasdermin E suppresses tumour growth by activating anti-tumour immunity. Nature.

[15] Bergsbaken T, Fink SL, Cookson BT (2009). Pyroptosis: host cell death and inflammation. Nat Rev Microbiol.

[16] Slack FJ, Chinnaiyan AM (2019). The role of non-coding RNAs in Oncology. Cell.

[17] Gandhi M, Caudron-Herger M, Diederichs S (2018). RNA motifs and combinatorial prediction of interactions, stability and localization of noncoding RNAs. Nat Struct Mol Biol.

[18] Derrien T, Johnson R, Bussotti G, Tanzer A, Djebali S, Tilgner H (2012). The GENCODE v7 catalog of human long noncoding RNAs: analysis of their gene structure, evolution, and expression. Genome Res.

[19] Carninci P, Kasukawa T, Katayama S, Gough J, Frith MC, Maeda N (2005). The transcriptional landscape of the mammalian genome. Science.

[20] Wang K, Jin W, Song Y, Fei X (2017). LncRNA RP11-436H11.5, competitive endogenous RNA, upregulates BCL-W expression by sponging miR-335–5p and promotes proliferation and invasion in renal cell carcinoma. Mol Cancer..

[21] Liu Y, Chen X, Che Y, Li H, Zhang Z, Peng W, Yang J (2022). LncRNAs as the regulators of brain function and therapeutic targets for Alzheimer's disease. Aging Dis.

[22] Li X, Zeng L, Cao C, Lu C, Lian W, Han J (2017). Long noncoding RNA MALAT1 regulates renal tubular epithelial pyroptosis by modulated miR-23c targeting of ELAVL1 in diabetic nephropathy. Exp Cell Res.

[23] Hu J, Wu H, Wang D, Yang Z, Dong J (2019). LncRNA ANRIL promotes NLRP3 inflammasome activation in uric acid nephropathy through miR-122-5p/BRCC3 axis. Biochimie.

[24] Py BF, Kim MS, Vakifahmetoglu-Norberg H, Yuan J (2013). Deubiquitination of NLRP3 by BRCC3 critically regulates inflammasome activity. Mol Cell.

[25] Yi H, Peng R, Zhang LY, Sun Y, Peng HM, Liu HD (2017). LincRNA-Gm4419 knockdown ameliorates NF-κB/NLRP3 inflammasome-mediated inflammation in diabetic nephropathy. Cell Death Dis..

[26] Xu F, Zhan X, Zheng X, Xu H, Li Y, Huang X (2020). A signature of immune-related gene pairs predicts oncologic outcomes and response to immunotherapy in lung adenocarcinoma. Genomics.

[27] Li X, Li Y, Yu X, Jin F (2020). Identification and validation of stemness-related lncRNA prognostic signature for breast cancer. J Transl Med.

[28] Xu F, Huang X, Li Y, Chen Y, Lin L (2021). m6A-related lncRNAs are potential biomarkers for predicting prognoses and immune responses in patients with LUAD. Mol Ther Nucleic Acids.

[29] He Y, Jiang Z, Chen C, Wang X (2018). Classification of triple-negative breast cancers based on Immunogenomic profiling. J Exp Clin Cancer Res.

[30] Zeng D, Ye Z, Wu J, Zhou R, Fan X, Wang G (2020). Macrophage correlates with immunophenotype and predicts anti-PD-L1 response of urothelial cancer. Theranostics.

[31] Hwang S, Kwon AY, Jeong JY, Kim S, Kang H, Park J (2020). Immune gene signatures for predicting durable clinical benefit of anti-PD-1 immunotherapy in patients with non-small cell lung cancer. Sci Rep.

[32] Tirosh I, Izar B, Prakadan SM, Wadsworth MH, Treacy D, Trombetta JJ (2016). Dissecting the multicellular ecosystem of metastatic melanoma by single-cell RNA-seq. Science (New York, NY).

[33] Pang C, Guan Y, Li H, Chen W, Zhu G (2016). Urologic cancer in China. Jpn J Clin Oncol.

[34] Yamamoto K, Ioroi T, Shinomiya K, Yoshida A, Harada K, Fujisawa M (2022). STAT3 polymorphism associates With mTOR inhibitor-induced interstitial lung disease in patients with renal cell carcinoma. Oncol Res.

[35] Funakoshi T, Lee CH, Hsieh JJ (2014). A systematic review of predictive and prognostic biomarkers for VEGF-targeted therapy in renal cell carcinoma. Cancer Treat Rev.

[36] Jonasch E, Walker CL, Rathmell WK (2021). Clear cell renal cell carcinoma ontogeny and mechanisms of lethality. Nat Rev Nephrol.

[37] Ding J, Wang K, Liu W, She Y, Sun Q, Shi J (2016). Pore-forming activity and structural autoinhibition of the gasdermin family. Nature.

[38] Broz P, Pelegrin P, Shao F (2020). The gasdermins, a protein family executing cell death and inflammation. Nat Rev Immunol.

[39] Yin Y, Li X, Sha X, Xi H, Li YF, Shao Y (2015). Early hyperlipidemia promotes endothelial activation via a caspase-1-sirtuin 1 pathway. Arterioscler Thromb Vasc Biol.

[40] Mamun AA, Wu Y, Nasrin F, Akter A, Taniya MA, Munir F (2021). Role of pyroptosis in diabetes and its therapeutic implications. J Inflamm Res.

[41] Jia C, Chen H, Zhang J, Zhou K, Zhuge Y, Niu C (2019). Role of pyroptosis in cardiovascular diseases. Int Immunopharmacol.

[42] Xi G, Gao J, Wan B, Zhan P, Xu W, Lv T (2019). GSDMD is required for effector CD8 T cell responses to lung cancer cells. Int Immunopharmacol..

[43] Zhang Z, Zhang Y, Xia S, Kong Q, Li S, Liu X (2020). Gasdermin E suppresses tumour growth by activating anti-tumour immunity. Nature.

[44] Wang Y, Gao W, Shi X, Ding J, Liu W, He H (2017). Chemotherapy drugs induce pyroptosis through caspase-3 cleavage of a gasdermin. Nature.

[45] Kumagai S, Togashi Y, Kamada T, Sugiyama E, Nishinakamura H, Takeuchi Y (2020). The PD-1 expression balance between effector and regulatory T cells predicts the clinical efficacy of PD-1 blockade therapies. Nat Immunol.

[46] Hou J, Zhao R, Xia W, Chang CW, You Y, Hsu JM (2020). PD-L1-mediated gasdermin C expression switches apoptosis to pyroptosis in cancer cells and facilitates tumour necrosis. Nat Cell Biol.

[47] Zhang P, Cao L, Zhou R, Yang X, Wu M (2019). The lncRNA Neat1 promotes activation of inflammasomes in macrophages. Nat Commun.

[48] Xu Y, Chen C, Liao Z, Xu P (2023). cGAS-STING signaling in cell death: mechanisms of action and implications in pathologies. Eur J Immunol.

[49] Zheng W, Feng D, Xiong X, Liao X, Wang S, Xu H, Le W, Wei Q, Yang L (2023). The role of cGAS-STING in age-related diseases from mechanisms to therapies. Aging Dis.

[50] Huang A, Zhou W (2023). Mn-based cGAS-STING activation for tumor therapy. Chin J Cancer Res.

[51] Ferro M, Musi G, Marchioni M, Maggi M, Veccia A, Del Giudice F (2023). Radiogenomics in renal cancer management-current evidence and future prospects. Int J Mol Sci.

[52] Feng S (2021). Gasdermins: making pores for pyroptosis. Nat Rev Immunol.

