# Supplementary material for: Development and validation of a kidney renal clear cell carcinoma prognostic model relying on pyroptosis-related LncRNAs-A multidimensional comprehensive bioinformatics exploration
Source: Eur J Med Res. 2023 Sep 12;28:341. doi: 10.1186/s40001-023-01277-2 (PMC10498568; doi:10.1186/s40001-023-01277-2)
Supplement: Supplementary file 1 — Additional file 1: Table S1. Gene names of the 52 pyroptosis-related genes. [file 40001_2023_1277_MOESM1_ESM.docx]

**GeneName**

BAK1

BAX

CASP1

CASP3

CASP4

CASP5

CHMP2A

CHMP2B

CHMP3

CHMP4A

CHMP4B

CHMP4C

CHMP6

CHMP7

CYCS

ELANE

GSDMD

GSDME

GZMB

HMGB1

IL18

IL1A

IL1B

IRF1

IRF2

TP53

TP63

AIM2

CASP6

CASP8

CASP9

GPX4

GSDMA

GSDMB

GSDMC

IL6

NLRC4

NLRP1

NLRP2

NLRP3

NLRP6

NLRP7

NOD1

NOD2

PJVK

PLCG1

PRKACA

PYCARD

SCAF11

TIRAP

TNF

GZMA
