# Supplementary material for: Development and validation of a kidney renal clear cell carcinoma prognostic model relying on pyroptosis-related LncRNAs-A multidimensional comprehensive bioinformatics exploration
Source: Eur J Med Res. 2023 Sep 12;28:341. doi: 10.1186/s40001-023-01277-2 (PMC10498568; doi:10.1186/s40001-023-01277-2)
Supplement: Supplementary file 2 — Additional file 2: Table S2. Names of the six lncRNAs in the model that are connected to pyroptosis. [file 40001_2023_1277_MOESM2_ESM.docx]

**LncRNA-Name**

LINC02747

LUCAT1

LINC00896

`KLHDC7B-DT`

LINC01138

LINC01671
