# Supplementary material for: Development and validation of a kidney renal clear cell carcinoma prognostic model relying on pyroptosis-related LncRNAs-A multidimensional comprehensive bioinformatics exploration
Source: Eur J Med Res. 2023 Sep 12;28:341. doi: 10.1186/s40001-023-01277-2 (PMC10498568; doi:10.1186/s40001-023-01277-2)
Supplement: Supplementary file 3 — Additional file 3: Table S3. In the risk model, the clinical characteristics are shared by the complete set, the training set, and the test set. [file 40001_2023_1277_MOESM3_ESM.docx]

Covariates Type Total Test Train Pvalue

Age <=65 348(65.66%) 177(67.05%) 171(64.29%) 0.5636

Age >65 182(34.34%) 87(32.95%) 95(35.71%)

Gender FEMALE 186(35.09%) 90(34.09%) 96(36.09%) 0.6957

Gender MALE 344(64.91%) 174(65.91%) 170(63.91%)

Grade G1 14(2.64%) 6(2.27%) 8(3.01%) 0.3819

Grade G2 227(42.83%) 104(39.39%) 123(46.24%)

Grade G3 206(38.87%) 111(42.05%) 95(35.71%)

Grade G4 75(14.15%) 37(14.02%) 38(14.29%)

Grade unknow 8(1.51%) 6(2.27%) 2(0.75%)

Stage Stage I 265(50%) 131(49.62%) 134(50.38%) 0.9977

Stage Stage II 57(10.75%) 29(10.98%) 28(10.53%)

Stage Stage III 123(23.21%) 61(23.11%) 62(23.31%)

Stage Stage IV 82(15.47%) 41(15.53%) 41(15.41%)

Stage unknow 3(0.57%) 2(0.76%) 1(0.38%)

T T1 271(51.13%) 133(50.38%) 138(51.88%) 0.9543

T T2 69(13.02%) 34(12.88%) 35(13.16%)

T T3 179(33.77%) 92(34.85%) 87(32.71%)

T T4 11(2.08%) 5(1.89%) 6(2.26%)

M M0 420(79.25%) 211(79.92%) 209(78.57%) 0.7995

M M1 78(14.72%) 41(15.53%) 37(13.91%)

M unknow 32(6.04%) 12(4.55%) 20(7.52%)

N N0 239(45.09%) 116(43.94%) 123(46.24%) 0.4103

N N1 16(3.02%) 10(3.79%) 6(2.26%)

N unknow 275(51.89%) 138(52.27%) 137(51.5%)
