# Supplementary figures and images for: Development and validation of a kidney renal clear cell carcinoma prognostic model relying on pyroptosis-related LncRNAs-A multidimensional comprehensive bioinformatics exploration
Source: Eur J Med Res. 2023 Sep 12;28:341. doi: 10.1186/s40001-023-01277-2 (PMC10498568; doi:10.1186/s40001-023-01277-2)

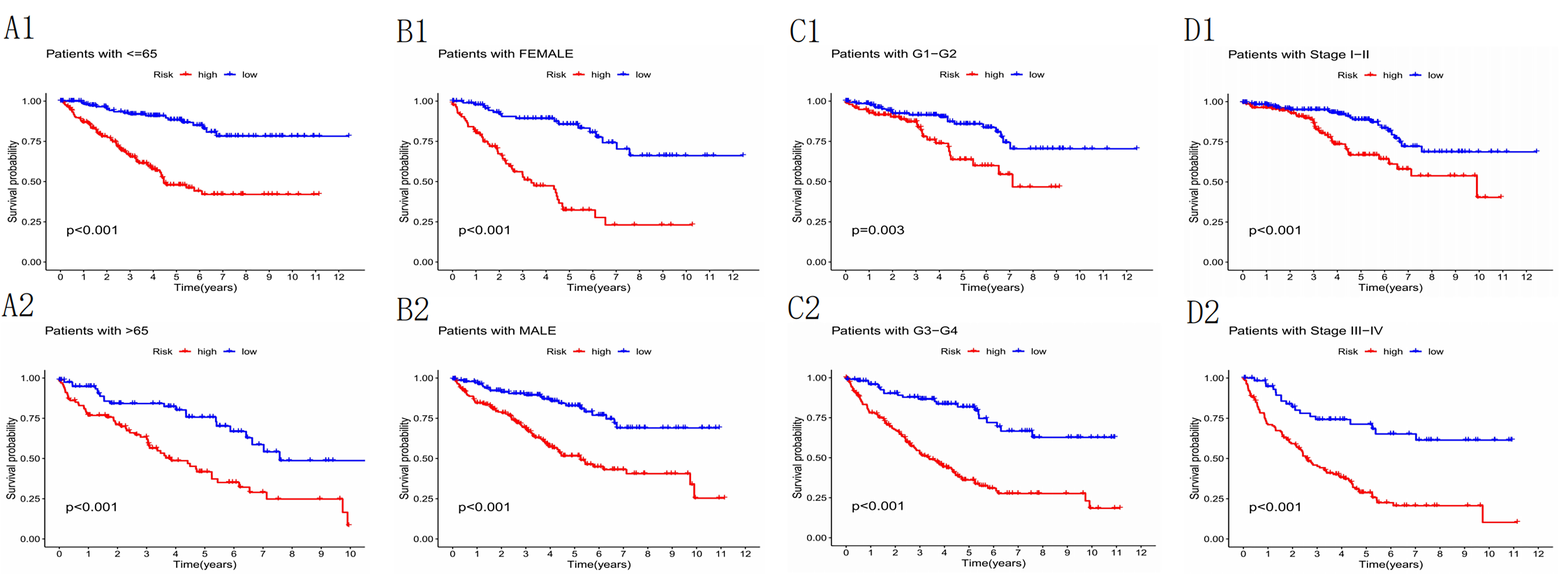

Supplement: Supplementary file 4 — Additional file 4: Figure S1. Kaplan‒Meier curves show differences in overall survival between the two groups in the entire TCGA set, stratified by age, sex, tumour grade, and stage. [file 40001_2023_1277_MOESM4_ESM.tif]

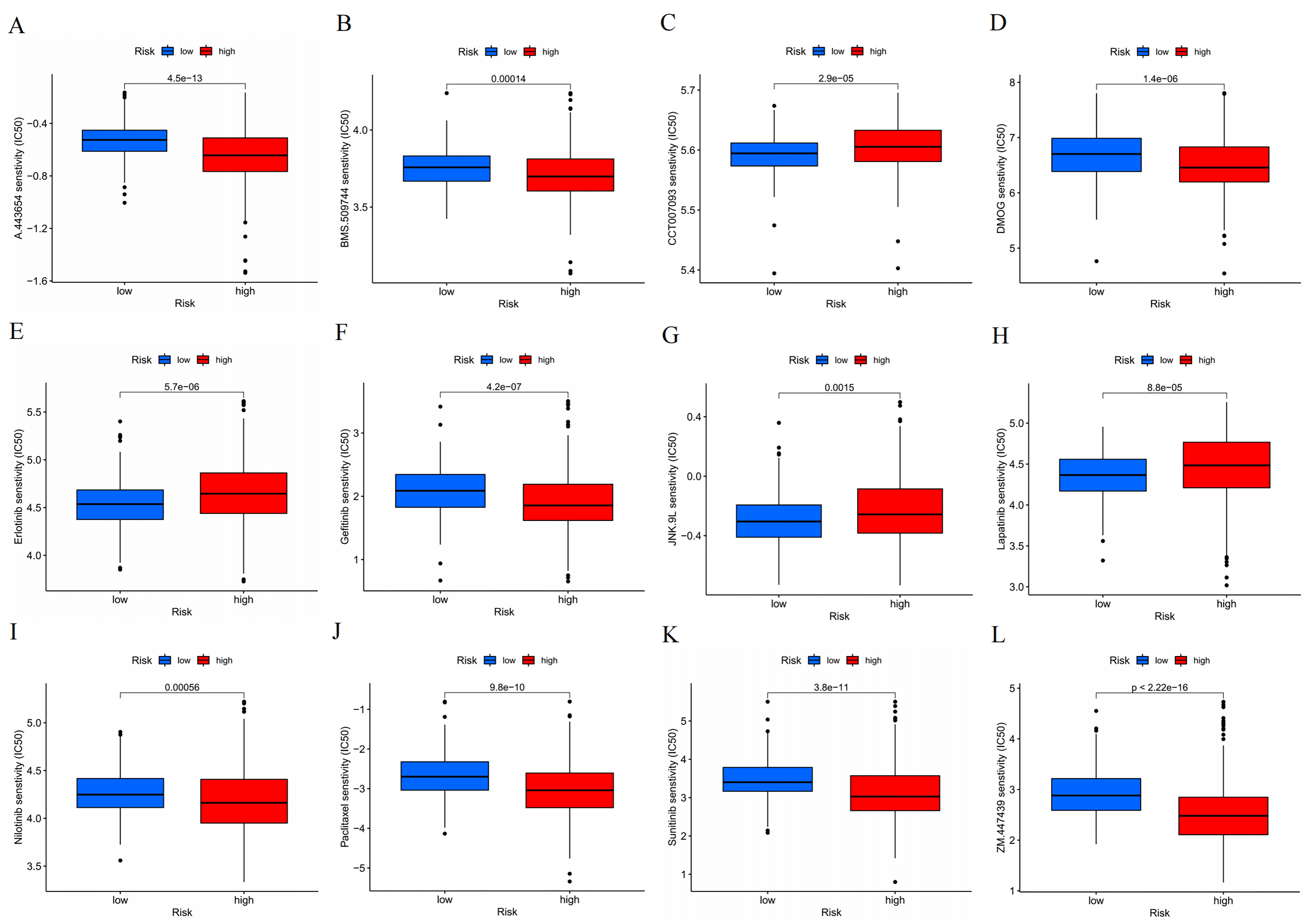

Supplement: Supplementary file 5 — Additional file 5: Figure S2. Compounds with partial sensitivity. [file 40001_2023_1277_MOESM5_ESM.tif]

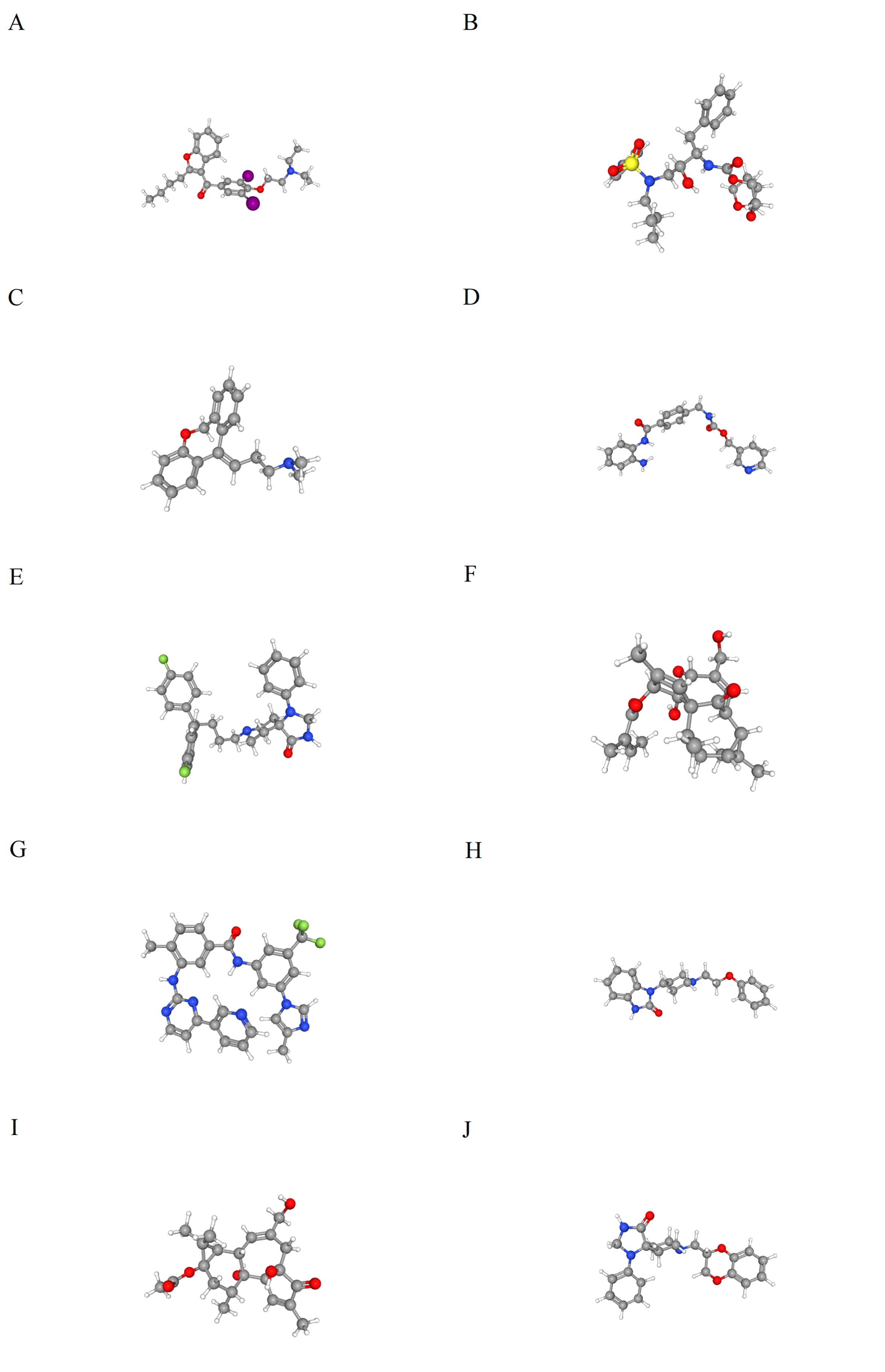

Supplement: Supplementary file 6 — Additional file 6: Figure S3. Three-dimensional structure of the top ten related drugs. [file 40001_2023_1277_MOESM6_ESM.tif]
